# Supplementary material for: Streptococcus pneumoniae promotes lung cancer development and progression
Source: iScience. 2023 Jan 4;26(2):105923. doi: 10.1016/j.isci.2022.105923 (PMC9852931; doi:10.1016/j.isci.2022.105923)
Supplement: Document S1. Figures S1–S3 and Tables S1–S3 [file mmc1.pdf]

iScience, Volume *26*

Supplemental information

Streptococcus pneumoniae promotes lung cancer development and progression

Ning Li, Huifen Zhou, Van K. Holden, Janaki Deepak, Pushpa Dhilipkannah, Nevins W. Todd, Sanford A. Stass, and Feng Jiang

Table S1. Spearman correlations between SP abundances in tumor tissue sand demographic and clinical characteristics of the NSCLC patients, related to Figure 6.

	Age	Gender	Race	Smoking status	Histological types	Stage
	R^2 (P value)	R^2 (P value)	R^2 (P value)	R^2 (P value)	R^2 (P value)	R^2 (P value)
SP abundances	0.239 (0.367)	0.116 (0.327)	0.184 (0.154)	0.239 (0.538)	0.085 (0.546)	0.684 (0.026)

R^2: correlation coefficient. The closer the absolute value of R2 is to 1, the greater the relativity.

Table S2. Univariate Cox Proportional Hazards regression analysis of covariates in relation to survival of patients, related to Figure 6.

Covariate	Overall survival
Age	0.036
Sex	0.6763
Smoking Status	0.148
Tumor histology	0.853
Stage	0.026
Abundance of *S. pneumoniae*	0.028
Expression of PAFR	0.039

The numbers in the table represent P values calculated with the Wald test.

P values <0.05 were considered statistically significant.

Table S3. Multivariate Cox proportional hazards regression analysis to evaluate the prognostic value of abundance of *S. pneumonia*e and clinical parameters, related to Figure 6.

Covariate	Overall survival
Age	0.039
Sex	0.564
Smoking Status	0.438
Tumor histology	0.462
Stage	0.029
Abundance of *S. pneumoniae*	0.027
Expression of PAFR	0.032

The numbers in the table represent P values calculated with the Wald test.

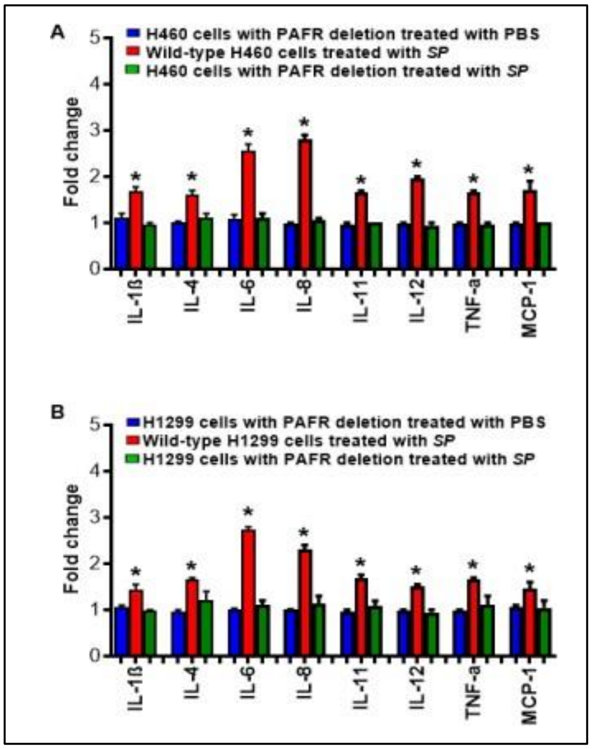

Figure S1. PCR array analysis of inflammatory cytokine gene expression in H460 and H1299 cells with deletion of PAFR, related to Figure 3.

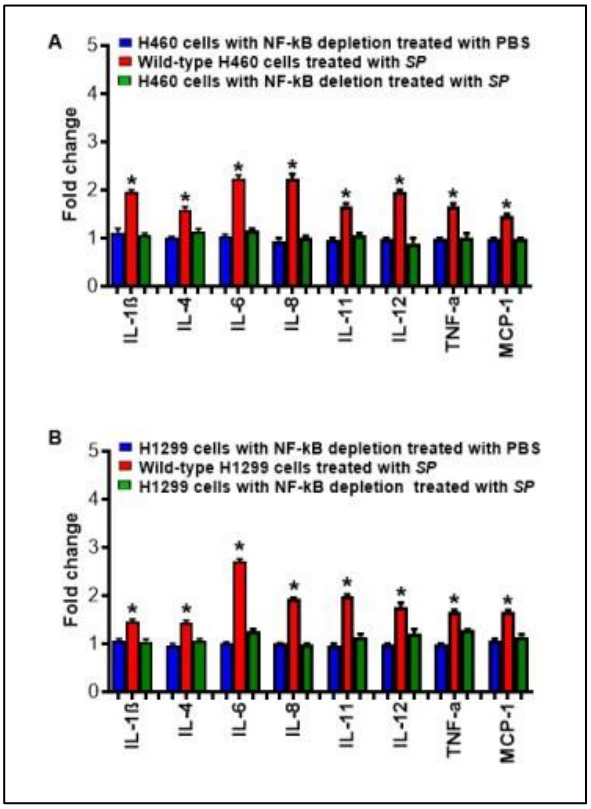

Figure S2. *SP* activated pro-inflammatory cytokines in the H460 and H1299 cells were inhibited by NF-kB depletion, related to Figure 3.

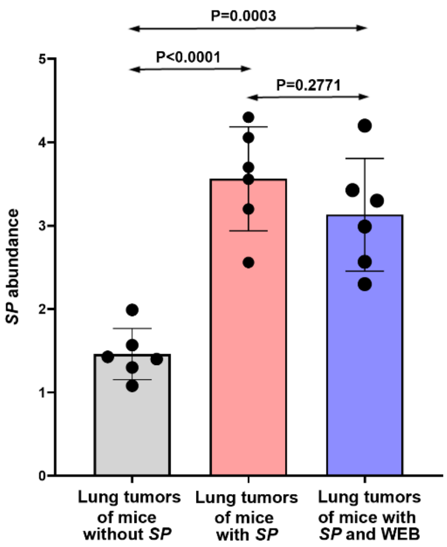

Figure S3. ddPCR analysis of *SP* abundance in lung tumors of NNK-mice with or without SP treatments or combined of SP and WEB2086, related to Figure 5.
